# Pain assessment and management practices in Hungarian neonatal intensive care units: a nationwide survey

**DOI:** 10.3389/fped.2026.1882635

**Published:** 2026-06-24

**Authors:** Johanna Ivancsó, Gyula Tálosi

**Affiliations:** 1 Neonatal Intensive Care Unit, Borsod-Abaúj-Zemplén County Hospital and University Teaching Hospital, Miskolc, Hungary; 2 Neonatal Intensive Care Unit, Bács-Kiskun County Teaching Hospital, Kecskemét, Hungary

**Keywords:** analgesia, neonatal intensive care unit, neonatal pain, neonate, preterm infant

## Abstract

**Introduction:**

Neonates hospitalized in Neonatal Intensive Care Units (NICUs) are frequently exposed to painful procedures. Despite increasing evidence regarding the adverse short- and long-term consequences of untreated neonatal pain, pain assessment and management remain inadequate worldwide. This study aimed to describe neonatal pain assessment and management practices in Hungarian level III NICUs.

**Methods:**

A prospective multicenter cross-sectional survey was conducted between June and October 2023. An anonymous online questionnaire was distributed to the heads of all Hungarian level III NICUs.

**Results:**

Fourteen of the 21 Hungarian level III NICUs participated in the survey (response rate: 67%). Only 2 (14%) of NICUs reported using a comprehensive written pain management guideline, and the same two (14%) NICUs routinely used validated neonatal pain assessment scales. Non-pharmacological pain management methods were used in all units, although their type and frequency varied substantially. Reported methods included kangaroo care, breastfeeding, expressed breast milk, pacifier, facilitated tucking, sensorial saturation, music therapy, massage, oral sucrose, and co-bedding. Sucrose administration and pacifier use were the most frequently reported non-pharmacological interventions (13/14, 93% each). Postoperative analgesia was routinely administered in all surgical NICUs, while procedural analgesia, in addition to continuous opioid administration was reported by only 40% of these centers. Routine analgesia during mechanical ventilation was used in 86% of units. The most frequently prescribed medications were paracetamol for postoperative pain and fentanyl for ventilated patients. One NICU reported the use of sedatives without concomitant analgesic therapy.

**Conclusion:**

Neonatal pain assessment and management practices remain inconsistent in Hungarian NICUs. These findings should be interpreted cautiously, as the study was exploratory and relied on self-reported practices, which may overestimate actual adherence to neonatal pain management recommendations. However, wider implementation of standardized guidelines, validated pain assessment tools, and evidence-based pain management strategies is warranted.

## 1 Introduction

Numerous studies have demonstrated that neonates, including preterm infants, are capable of perceiving and responding to pain. During hospitalization in Neonatal Intensive Care Units (NICUs), newborns are routinely exposed to painful procedures. A prospective observational study performed in a Hungarian NICU reported that during the first 14 days of hospitalization, each neonate underwent a mean of 93 painful interventions, corresponding to an average of 8.2 painful procedures per day, with 68.5% performed without analgesia (1). Similar findings have been reported internationally (2).

Evidence suggests that repeated and untreated neonatal pain may adversely affect neurodevelopment, stress responses, cognitive function, learning abilities, and long-term health outcomes (3). Despite increasing awareness and the availability of international recommendations (4–7), neonatal pain remains under-assessed and undertreated worldwide (8–11).

Hungary is located in Central Europe. Evidence from a review of studies conducted across 18 European countries, which did not include Hungary, has demonstrated substantial variability in sedation and analgesia practices both between neonatal intensive care units and across countries (12). Similarly, a recent study from Italy reported marked heterogeneity in neonatal analgesia practices among neonatal intensive care units (13).

A landmark international study published in early 2024 concluded that a country's socio-economic status, measured via the Sociodemographic Index (SDI), is strongly associated with the quality, availability, and standardization of newborn pain management (14). Hungary is classified as a high-SDI country; however, to the best of our knowledge, neonatal pain management practices have not previously been investigated at a national level in Hungary.

This study aimed to describe current neonatal pain assessment and management practices in Hungarian level III NICUs. The study addressed three key domains of neonatal pain management: (1) the availability and implementation of written pain management protocols, (2) the use of validated neonatal pain assessment tools, and (3) the application of pharmacological and non-pharmacological interventions for postoperative pain and for mechanically ventilated neonates in neonatal intensive care units.

## 2 Methods

### 2.1 Design

A prospective multicenter cross-sectional survey was conducted between June and October 2023 in Hungarian level III NICUs. The study aimed to assess level III NICUs; therefore, level II NICUs were excluded.

An anonymous online questionnaire was developed following a review of the relevant literature and previously published neonatal pain management surveys. The development, administration, and reporting of the questionnaire were guided by the Checklist for Reporting Results of Internet E-Surveys (CHERRIES) framework (15). The questionnaire consisted of 29 single- and multiple-choice questions and was created using Google Forms. To assess content validity, the draft questionnaire was reviewed by five experts with experience in the field. The experts evaluated the relevance, clarity, and comprehensibility of the items and provided qualitative feedback. Based on their comments, several items were revised to improve wording and content coverage. Subsequently, a pilot test was conducted among three senior neonatologists who did not participate in the study to assess comprehensibility, feasibility, and completion time. Based on this, no additional changes were needed.

The questionnaire included five domains: (A) use of written pain management guidelines; (B) pain assessment practices; (C) non-pharmacological pain management; (D) pharmacological pain management for postoperative and mechanically ventilated neonates; and (E) self-evaluation of pain management practices (Supplementary Appendices 1, 2).

### 2.2 Participant

At the time of the study, there were 21 tertiary-level NICUs in Hungary. The survey link was distributed via e-mail to the heads of all Hungarian level III NICUs. In a cover letter, all department heads were informed of the questionnaire's objective, the importance of anonymity, and the researcher's contact information. Two reminders were sent, one month apart.

As the data were based on self-reports from NICU heads, the results may be affected by reporting and social desirability bias.

### 2.3 Data collection

Only minimal demographic data were collected to preserve anonymity. Participation was voluntary, and submission of the completed questionnaire was considered informed consent.

Because the questionnaire was distributed exclusively to NICU directors and each participating unit was asked to submit a single completed response, the likelihood of duplicate data entries was considered minimal.

No missing data were observed; therefore, all returned questionnaires were included in the final analysis.

### 2.4 Ethical considerations

The study involved an anonymous survey of healthcare professionals and did not include patient-level data or clinical intervention; therefore, in accordance with Act CLIV of 1997 on Health Care and the applicable Hungarian regulations on human biomedical research, formal ethics committee approval was not required (16).

### 2.5 Data analysis

Data were analysed using descriptive and inferential statistical methods. Categorical variables were summarised as frequencies and percentages. Given the small sample size and the non-normal distribution of the data, non-parametric tests were applied for group comparisons. Differences between NICUs using a pain management guideline and those not using a guideline were assessed using the Mann–Whitney *U* test. All statistical tests were two-tailed, and a *p*-value of <0.05 was considered statistically significant.

Due to the limited number of participating NICUs, the analyses were considered exploratory, and the results should be interpreted with caution regarding statistical power and generalisability.

## 3 Results

Twenty-one units were asked to participate, and fourteen questionnaires were returned, corresponding to a response rate of 67%. No questionnaires were excluded from analysis.

### 3.1 Written guidelines

Only two (2/14) NICUs (14%) reported using a comprehensive written pain management guideline, which includes recommendations for reducing the number of painful interventions, assessing pain, and non-pharmacological and pharmacological pain relief techniques. In five (5/12) departments (36%) guidelines were used to manage the pain of only certain procedures (venipuncture, lumbar puncture, heel prick, intubation, chest drain insertion).

### 3.2 Pain assessment

Routine use of validated neonatal pain assessment scales was reported in two (2/14) NICUs (14%). The Astrid Lindgren and Lund Children's Hospitals Pain and Stress Assessment Scale for Preterm and Sick Newborn Infants (ALPS Neo) was used in one department, while the Premature Infant Pain Profile (PIPP), and the Behavioral Indicators of Infant Pain (BIIP) scales were used in the other department. In one center, pain assessment scales were used for both procedural and postoperative pain evaluation (PIPP and BIIP). In the other department the use of a pain scale was limited to painful procedures (ALPS Neo). Pain scoring was performed by physicians and nurses; however, only one center documented pain scores in the medical record. No unit involved parents in pain assessment.

Among centers not using validated pain scales (12/14, 86%), the most commonly reported reasons were reliance on alternative clinical signs (10/12, 83%), lack of human resources (5/12, 42%), absence of an appropriate scale (3/12, 25%), perceived complexity of use (2/12, 17%), and time constraints (1/12, 8%). Units not using formal pain scales relied on physiological and behavioral indicators to assess pain (12/12, 100%).

### 3.3 Non-pharmacological pain management

All responding NICUs reported using at least three non-pharmacological pain management strategies (Table 1). However, both the number and type of interventions varied considerably among centers. Reported methods included kangaroo care, breastfeeding, expressed breast milk, pacifiers, facilitated tucking, sensorial saturation, music therapy, massage, oral sucrose, and co-bedding. Among the 14 participating NICUs, sucrose administration and pacifier use were the most frequently reported non-pharmacological interventions (13/14, 93% each), followed by kangaroo care (12/14, 86%). Breastfeeding and expressed breast milk were used in 71% (10/14) of units, while facilitated tucking was reported by 57% (8/14). Music therapy was less common (5/14, 36%), and co-bedding and massage were each reported by 21% (3/14) of NICUs. Sensorial saturation was rarely used (1/14, 7%).

**Table 1:** Number and type of non-pharmacological pain management methods used in Hungarian NICUs. NICU 13. and 14. use a comprehensive written pain management guideline and validated pain scales.

NICU	Sucrose	Pacifier	Kangaroo care	Breast feeding	Expressed breast milk	Facilitated tucking	Music therapy	Co-bedding	Massage	Sensorial saturation	Number of all methods
1. NICU	x	x					x				3
2. NICU	x	x	x	x							4
3. NICU	x	x	x			x					4
4. NICU	x	x				x			x		4
5. NICU		x	x	x	x						4
6. NICU	x		x	x	x			x			5
7. NICU	x	x	x	x	x						5
8. NICU	x	x	x	x	x		x				6
9. NICU	x	x	x	x	x	x					6
10. NICU	x	x	x		x	x	x				7
11. NICU	x	x	x	x	x	x				x	7
12. NICU	x	x	x	x	x	x	x		x		8
13. NICU	x	x	x	x	x	x	x	x			8
14. NICU	x	x	x	x	x	x		x	x		8
% of NICUs	93%	93%	86%	71%	71%	57%	36%	21%	21%	7%	

Ten NICUs (10/14, 71%) did not routinely document non-pharmacological interventions in medical records. Four units (4/14, 29%) documented only oral sucrose administration.

### 3.4 Postoperative pain management

Surgical cases were managed in five (5/14, 36%) NICUs, all of which reported the use of postoperative analgesia (5/5, 100%). However, only 40% (2/5) used additional procedural analgesia during continuous intravenous opioid administration.

The most frequently prescribed medications for postoperative pain management were paracetamol (5/5, 100%), nalbuphine (4/5, 80%), and fentanyl (4/5, 80%). Smaller proportions of centers used ibuprofen (2/5 40%), morphine (1/5, 20%), ketamine (1/5, 20%), and metamizole (1/5, 20%).

### 3.5 Mechanical ventilation

**Figure 1:**
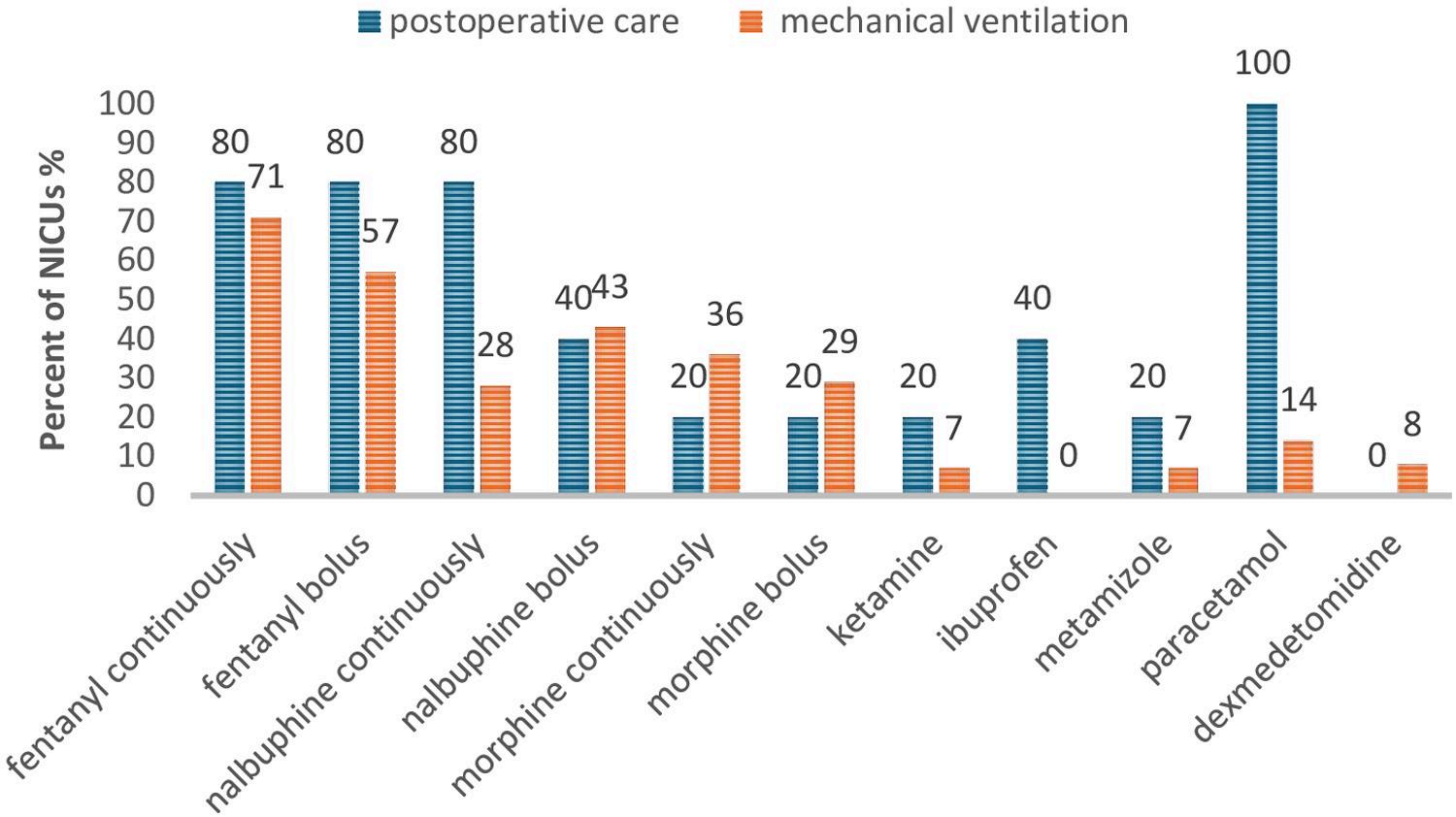
Analgesic medication used during mechanical ventilation and postoperatively in Hungarian NICUs.

Routine analgesia for mechanically ventilated neonates was reported by 86% of centers (12/14). Fentanyl was the most frequently used analgesic (10/14, 71%), followed by nalbuphine (6/14, 43%) and morphine (5/14, 36%) (Figure 1).

### 3.6 Sedative use

One NICU reported the use of sedatives, including thiopental, chloral hydrate, and benzodiazepines, without concomitant analgesic therapy.

### 3.7 Self-evaluation of pain management

None of the respondents reported complete satisfaction with pain management practices in their department. Eleven neonatologists (79%) considered their local practice acceptable but improvable.

### 3.8 Comparison of NICUs

The two NICUs using a comprehensive written pain management guideline showed concordance with the two units applying a validated pain assessment scale. These units were characterized by a higher reported use of non-pharmacological pain management strategies, as well as a broader range of pharmacological analgesic agents used in clinical practice.

Given the limited number of guideline-using units (*n* = 2) and non-guideline-using units (*n* = 12), formal statistical comparisons were not performed due to insufficient statistical power.

NICUs using complex pain management guidelines and procedural pain management guidelines (*n* = 7) reported a lower mean number of non-pharmacological methods compared with units (*n* = 7) not using a guideline (5.0 vs. 6.3 methods). However, the difference was not statistically significant (Mann–Whitney *U* test, *p* = 0.216). There was no statistically significant difference in the number of analgesic medications used between NICUs that used a pain management guideline and those that did not (2,1 vs. 1,8; Mann–Whitney *U* test, *p* = 0.79).

Due to the limited number of surgical centers included in the survey, statistical power was insufficient to support formal comparisons between surgical and non-surgical units.

## 4 Discussion

This cross-sectional nationwide survey described neonatal pain management practices in Hungarian tertiary-level Neonatal Intensive Care Units. To our knowledge, this is the first such study in Hungary. The findings indicate that neonatal pain management is inconsistent in Hungarian NICUs.

Detailed written guidelines were lacking in 86% of Hungarian NICUs. In the absence of written recommendations, pain treatment is provided on an individual basis. In an Italian survey, the use of written guidelines led to a twelve-fold increase in the treatment of acute pain and an eight-fold increase in the likelihood of analgesia and sedation being used for prolonged pain and discomfort (11). The use of the guideline led to sustainable long-term improvements in pain management practices (13). International guidelines recommend that all NICUs have neonatal pain management policies (6, 17, 18). The two Hungarian NICUs using a comprehensive written pain management guideline and a validated pain assessment scale were characterized by a higher reported use of non-pharmacological pain management strategies, as well as a broader range of pharmacological analgesic agents. Given the limited number of guideline-using units, statistical comparisons were not performed due to insufficient statistical power. The wider adoption of written guidelines in Hungarian NICUs is strongly recommended. Recent evidence suggests that the main barriers to implementing neonatal pain assessment and management protocols include organizational, professional, tool-related and national factors (11, 13, 14, 19, 20). Organizational barriers include high workload, insufficient nurse-to-patient ratios, time constraints, excessive documentation requirements, institutional culture, and the lack of standardized protocols for routine pain assessment and management. Professional barriers involve limited education and training, and resistance to changing established clinical practices. Tool-related barriers include the absence of a universally accepted gold-standard scale, the complexity of assessing pain in non-verbal preterm infants, variability in pain expression according to gestational age and clinical condition, and the limited applicability of some scales in ventilated, sedated, or critically ill neonates. Furthermore, difficulties in distinguishing pain from stress or other sources of discomfort may reduce confidence in pain assessment tools and contribute to their inconsistent use in clinical practice. National barriers are the low socio-economic status of the country and the national policy. Most Hungarian units (86%) did not use validated neonatal pain scales. Two units used a total of three types of pain scales (PIPP, ALPS Neo, BIIP). Pain scores were documented in only one unit, and none of the NICUs involved parents in pain assessment. According to systematic literature research, numerous neonatal pain scales have been published with different levels of validity and reliability (21, 22). A multidisciplinary European Society of Pediatric and Neonatal Intensive Care (ESPNIC) position statement widely recommends using age-appropriate pain assessment tools (23). In Hungarian NICUs, the ALPS Neo scale was used for assessing procedural pain, while the PIPP and BIIP scales were used for procedural and postoperative pain assessment. However, these instruments were originally developed for different clinical contexts: the BIIP assesses acute procedural pain in preterm infants, the PIPP is designed to evaluate procedural pain in both preterm and term infants, whereas the ALPS-Neo scale has been validated for the assessment of prolonged pain in preterm and term neonates. Our findings suggest that pain assessment tools in Hungarian NICUs are not always used in accordance with their original validation purposes. The reported use of these instruments across different clinical contexts may indicate a mismatch between the type of pain being assessed and the intended application of the assessment tool, potentially affecting the validity and reliability of pain evaluation. The authors of a systematic review recommend using scales that have been validated for construct validity, internal consistency, and inter-rater reliability. Scales with lower risk of bias are COMFORT, Échelle Douleur Inconfort Nouveau-Né (EDIN), Evaluation Enfant Douleur (EVENDOL), Neonatal Facial Coding System (NFCS), Neonatal Pain, Agitation and Sedation Scale (N-PASS), and PIPP (22). Among these, PIPP was used in one Hungarian NICU. Documented pain score is associated with higher use of pain-reducing interventions (10). Research has shown that effective pain management methods were more likely to be used in newborns when mothers were present during painful procedures, highlighting the positive impact of parental presence (24). Parental education has been shown to improve pain management practices (25). Evidence indicates that parent-delivered pain management strategies, such as skin-to-skin contact, breastfeeding, and parental live singing are effective in alleviating procedural pain among term and preterm infants. Nevertheless, parental involvement in pain assessment, parent-targeted education, and interprofessional training are all underutilized, identifying these areas as priorities for quality improvement (26). Family-centered care is increasingly recognized as an important component of neonatal pain management, and greater parental involvement may represent an opportunity for future improvements in pain management practices.

The use of non-pharmacological pain management therapies was widespread in all Hungarian NICUs, although their variable use was unit-dependent. Oral sucrose was the most frequently used non-pharmacological therapy. However, effective methods proven by available evidence, such as breastfeeding, kangaroo care, expressed breast milk, and sensorial saturation were less common. Non-pharmacological therapies are recommended as the first step in neonatal pain relief, due to their favorable side effect profile and beneficial long-term effects (27). Education and efforts to eliminate barriers can improve their use. Giving sucrose for pain relief was poorly documented, according to our findings. Sucrose was found to be effective in reducing behavioural response to procedural pain, but additional research is needed to determine the minimally effective dose, the effect of repeated doses on pain intensity and long-term neurodevelopmental outcomes (28). Current evidence supports the efficacy of sucrose for procedural pain, although uncertainties remain regarding the long-term effects of repeated exposure (29). It is recommended to document each dose of sucrose, indicating a maximum total dose that can be given every 24 h (30).

A notable finding of our survey was that 71% of Hungarian NICUs did not routinely document the use of non-pharmacological pain-relief interventions, potentially leading to an underestimation of their actual implementation in clinical practice. Accurate documentation is essential for quality improvement initiatives, clinical audits, and monitoring adherence to pain management protocols. Failure to record non-pharmacological measures may lead to underestimation of their actual use and limits the ability to evaluate their effectiveness, consistency, and contribution to multimodal pain management strategies. Improving documentation practices may therefore represent an important step toward optimizing neonatal pain management and supporting evidence-based care.

According to our study, the use of analgesia for postoperative pain management (100%) and for mechanically ventilated newborns (86%) was common in Hungarian NICUs. However, only 40% of units using continuous opioid infusion for postoperative pain used procedural analgesia in addition. Mechanical ventilation is one of the most common sources of chronic pain in Neonatal Intensive Care Units. The routine use of opioids is not supported by current evidence. Opioid therapy should be used selectively, based on individual clinical assessment (5, 31). Continuous morphine administered for other reasons in preterm infants does not provide adequate analgesia for procedural pain. In these cases, other targeted analgesic therapy is necessary (32). Therefore, special attention should be paid to procedural pain relief in preterm infants receiving continuous opioid therapy. Evidence comparing continuous systemic opioid infusion with intermittent bolus administration for postoperative pain management in neonates remains limited. A recent review found insufficient evidence to determine whether continuous opioid infusion provides superior pain relief compared with intermittent bolus dosing. Furthermore, none of the included studies reported key long-term outcomes, such as all-cause mortality during initial hospitalization, significant neurodevelopmental impairment, or cognitive and educational outcomes beyond five years of age (31).

In addition to paracetamol, a small proportion of participating NICUs reported the use of ibuprofen, metamizole, and ketamine for postoperative pain management. Although these agents were used infrequently, their inclusion reflects a growing interest in multimodal analgesic strategies aimed at improving pain control while minimizing opioid exposure. Opioid-sparing approaches have gained increasing attention in neonatal care because of concerns regarding opioid-related adverse effects, including respiratory depression, tolerance, withdrawal, and potential impacts on the developing brain (33). Nonsteroidal antiinflammatory drugs (NSAIDs) are well-established components of multimodal postoperative analgesia in older children and adults. In neonates, however, evidence remains considerably more limited. NSAIDs may reduce pain and decrease opioid requirements, but concerns persist regarding their safety profile, particularly the risks of renal impairment, gastrointestinal complications, and platelet dysfunction with a potential increase in bleeding tendency. Furthermore, neonatal-specific pharmacokinetic and pharmacodynamic data for analgesic indications are scarce, and most available experience with ibuprofen relates to pharmacological closure of patent ductus arteriosus rather than postoperative pain management (5). One participating NICU also reported the use of ketamine. Ketamine has attracted increasing interest in neonatal intensive care because it provides analgesia and sedation while generally preserving cardiovascular stability and causing less respiratory depression than opioids. These characteristics may be advantageous in postoperative neonates, particularly those who are hemodynamically vulnerable. Nevertheless, the evidence supporting routine ketamine use for postoperative pain management in neonates remains limited. Important uncertainties persist regarding optimal dosing regimens, duration of therapy, and long-term neurodevelopmental effects. Recent reviews emphasize that although ketamine and NSAIDs represent promising adjunctive agents within opioid-sparing strategies, further neonatal-specific studies are required before their widespread implementation can be recommended for postoperative analgesia (5). Metamizole was reported by one participating NICU despite its heterogeneous regulatory status worldwide. While the drug is banned or not licensed in several countries, including the United States, the United Kingdom, and Scandinavia, it continues to be widely used in many European countries and has been incorporated into pediatric postoperative pain management guidelines in Germany, Austria, and European consensus recommendations. The principal safety concern associated with metamizole is agranulocytosis, particularly during prolonged treatment or use beyond approved indications. Nevertheless, current evidence indicates that short-term perioperative administration is generally well tolerated and associated with a low risk of severe adverse events, although neonatal-specific data remain limited and further research is warranted (34). Overall, the limited use of NSAIDs, metamizole, and ketamine observed in our survey likely reflects the balance between the desire to implement multimodal analgesia and the current lack of high-quality neonatal-specific evidence. Future studies should focus on defining the efficacy, safety, and opioid-sparing potential of these agents in postoperative neonatal care to support the development of evidence-based analgesic protocols.

One Hungarian NICU used sedatives alone for pain relief. These medications do not relieve pain but can suppress the pain response; therefore, they should not be used alone as substitutes for analgesics. However, adjunctive sedation may be required in some cases to achieve adequate comfort (5). These findings further underscore the importance of distinguishing analgesia from sedation in neonatal care. The reported use of sedatives as part of pain management strategies may therefore indicate ongoing gaps in knowledge or inconsistencies in clinical practice. Addressing these issues through targeted education, multidisciplinary training programs, and the implementation of standardized, evidence-based protocols may improve pain recognition and promote more effective and appropriate analgesic management in neonates.

All responding senior neonatologists acknowledged inadequate pain management in their departments but had not yet adopted formal protocols or guidelines. Previous international research has reported a relationship between a country's SDI and the quality and implementation of neonatal pain management practices (14). Although this analysis has demonstrated an association between lower SDI and inadequate neonatal pain management, our findings suggest that this relationship is not linear at the national level. Hungary is classified as a high-SDI country; therefore, the inadequate neonatal pain management practices identified in our survey cannot be readily attributed to limited socioeconomic development. Our findings suggest that factors beyond national SDI may play a more important role in determining neonatal pain management practices. Consequently, high SDI does not necessarily ensure the use of evidence-based neonatal pain assessment and management guidelines. Future implementation efforts may benefit from the application of established implementation science frameworks. The Knowledge-to-Action framework could support the systematic translation of evidence into clinical practice, while the Consolidated Framework for Implementation Research (CFIR) may help identify and address contextual factors influencing the adoption of neonatal pain assessment and management protocols (35, 36).

The findings of this survey informed the development of a national neonatal pain management guideline, which has since been presented at a national congress and made publicly available.

This study has several limitations. Self-report questionnaires are vulnerable to bias from fixed or extreme responses, fear of losing anonymity, or social desirability. To preserve the anonymity of participating NICUs, detailed center-specific data were not collected, limiting our ability to explore differences between units. Responses from NICU heads may not reflect actual bedside practice and are based on their perceptions of current management rather than observed behavior. Another limitation is the relatively small sample size, which may limit the generalizability of the findings, reduce statistical power, and decrease the ability to detect between-group differences. Nevertheless, the results provide the first nationwide overview of neonatal pain management practices in Hungarian NICUs.

## 5 Conclusion

The findings provide valuable baseline data on current neonatal pain management practices, but should not be interpreted as a definitive measure of care quality across Hungarian neonatal intensive care units. Our results demonstrate that despite increasing awareness of the adverse consequences of untreated neonatal pain, pain assessment and management practices remain inconsistent in Hungarian NICUs. Further evaluation is needed to determine the barriers to appropriate neonatal pain management. Broader implementation of the new national neonatal pain management guideline, validated pain assessment tools, and evidence-based pain management strategies should be encouraged.

## Data Availability

The raw data supporting the conclusions of this article will be made available by the authors, without undue reservation.
