# Supplementary material for: Pain assessment and management practices in Hungarian neonatal intensive care units: a nationwide survey
Source: Front Pediatr. 2026 Jun 24;14:1882635. doi: 10.3389/fped.2026.1882635 (PMC13341843; doi:10.3389/fped.2026.1882635)
Supplement: Supplementary file 1 [file Table1.docx]

Does your unit care for surgical cases (pre- and postoperative care)?

- yes
- no

Is there any written guideline regarding pain management in the unit?

- yes
- no

If yes, what is the nature of this guideline? (multiple answers possible)

- comprehensive: includes recommendations to reduce painful procedures, pain assessment, and pharmacological and non-pharmacological pain management options
- pain management guideline for certain procedures (e.g., LISA, intubation, etc.)
- guideline for postoperative pain management
- pain management guideline for mechanically ventilated patients
- other: ______

Is there a written guideline for pain management related to procedures (e.g., intubation)?

- yes
- no

If yes, for which procedures is there a written pain management guideline? (multiple answers possible)

- heel stick
- venipuncture
- ophthalmologic examination (retinopathy of prematurity screening)
- lumbar puncture
- intubation
- chest drain insertion
- central venous catheter insertion
- Less Invasive Surfactant Administration (LISA)
- other: ______

Is pain assessed using any method in the unit?

- yes
- no

Is a validated pain scale used in the unit?

- yes
- no

If yes, in what situations? (multiple answers possible)

- during painful procedures
- in postoperative cases
- for research purposes
- only when analgesia is applied
- routinely, according to protocol
- routinely, but there is no protocol for its use
- other: ______

Who performs pain score assessment? (multiple answers possible)

- physician
- nurse
- parent
- other: ______

Is the assessed pain score documented?

- yes
- no

How frequently is pain assessment performed? (multiple answers possible)

- at least once per shift
- before and after invasive procedures
- Immediately after surgery and hourly until analgesia is optimal
- every 4 hours during continuous analgesia
- other: ______

Which pain scale(s) are used?

______

If no pain scale is used, why not? (multiple answers possible)

- no appropriate scale available
- too time-consuming
- too complicated
- not considered important
- pain can be adequately managed without it
- rely on other clinical signs (physical, behavioral)
- measured by other methods (e.g., NIRS, NIPE)
- lack of human resources
- other: ______

If pain is assessed using other methods, which ones? (multiple answers possible)

- NIRS
- amplitude-integrated EEG
- NIPE monitor
- physiological and/or behavioral changes
- other: ______

If pain is assessed based on physiological and behavioral signs, what is evaluated? (multiple answers possible)

- heart rate
- blood pressure
- respiratory rate
- oxygen saturation
- muscle tone
- crying
- facial expression
- other: ______

Are non-pharmacological pain relief methods used in the unit?

- yes
- no

If yes, which methods are used? (multiple answers possible)

- oral sucrose solution
- breastfeeding
- expressed breast milk
- pacifier/non-nutritive sucking
- kangaroo care / skin-to-skin contact
- massage
- multisensory stimulation
- music therapy
- aromatherapy
- co-bedding for twins
- facilitated tucking/positioning
- other: ______

If used, are non-pharmacological methods documented in medical records?

- yes
- no

If yes, which methods are documented? (multiple answers possible)

- oral sucrose solution
- breastfeeding
- expressed breast milk
- pacifier/non-nutritive sucking
- kangaroo care / skin-to-skin contact
- massage
- multisensory stimulation
- music therapy
- aromatherapy
- co-bedding for twins
- facilitated tucking/positioning
- other: ______

If non-pharmacological methods are not used, why not? (multiple answers possible)

- in case of pain pharmacological analgesia is used
- ineffective
- not considered important
- not available
- forgotten in practice
- short procedures make it unnecessary
- other: ______

Is routine analgesia used for mechanically ventilated (intubated) patients in the unit?

- yes
- no

If yes, what is used? (multiple answers possible)

- morphine continuous infusion
- morphine bolus
- fentanyl continuous infusion
- fentanyl bolus
- nalbuphine continuous infusion
- nalbuphine bolus
- ketamine
- other: ______

Is routine postoperative analgesia used in the unit?

- yes
- no
- no postoperative patients are treated

If yes, what is used? (multiple answers possible)

- morphine continuous infusion
- morphine bolus
- fentanyl continuous infusion
- fentanyl bolus
- nalbuphine continuous infusion
- nalbuphine bolus
- ketamine
- paracetamol
- ibuprofen
- other: ______

Do patients receiving continuous postoperative analgesia receive additional analgesia for procedural pain (e.g., venipuncture)?

- yes
- no
- no postoperative patients are treated

Are sedatives (e.g., phenobarbital) used for pain management without analgesia?

- yes
- no

If yes, which ones? (multiple answers possible)

- benzodiazepines
- phenobarbital
- chloral hydrate
- other: ______

Do you consider the pain management practice on the unit appropriate?

- yes
- no
- not bad, but could be improved
- other: ______

Any other comments, observations, or remarks regarding the survey or pain management practice on the unit:

______
