# Supplementary material for: Pain assessment and management practices in Hungarian neonatal intensive care units: a nationwide survey
Source: Front Pediatr. 2026 Jun 24;14:1882635. doi: 10.3389/fped.2026.1882635 (PMC13341843; doi:10.3389/fped.2026.1882635)
Supplement: Supplementary file 2 [file Table2.docx]

Látnak-e el sebészeti eseteket (közvetlen pre- és posztoperatív ellátás)?

- igen
- nem

Van-e az osztályon bármilyen, fájdalomcsillapítással kapcsolatos írásos protokoll?

- van
- nincs

Ha van, milyen jellegű ez a protokoll? (több válasz is megjelölhető)

- komplex: a fájdalmas beavatkozások számát csökkentő ajánlással, fájdalomméréssel, gyógyszeres és nem gyógyszeres fájdalomcsillapítási lehetőségekkel
- bizonyos bevatkozások (pl. LISA, intubálás, stb) fájdalmának csillapításához van protokoll
- a posztoperatív fájdalomcsillapításról van protokoll
- a tartósan lélegeztetett betegek fájdalmának csillapításáról van protokoll
- egyéb:

Van-e írásos protokoll a különböző beavatkozásokhoz (pl. intubálás) kapcsolódó fájdalomcsillapításról?

- van
- nincs

Ha van, mely beavatkozások fájdalomcsillapításáról van írásos protokoll? (több válasz is megjelölhető)

- sarokszúrás
- vénaszúrás
- szemészeti vizsgálat (retinopathia prematurorum szűrés)
- lumbálpunkció
- intubálás
- mellkasi drén behelyezése
- centrális vénás kanül behelyezése
- Less Invasive Surfactant Administration (LISA)
- egyéb:

Mérik-e az újszülöttek, koraszülöttek fájdalmát bármilyen módszerrel?

- igen
- nem

Használnak-e az osztályon validált fájdalomskálát?

- igen
- nem

Ha igen, milyen helyzetekben? (több válasz is megjelölhető)

- fájdalmas beavatkozásoknál
- posztoperatív esetekben
- kutatási célokból
- csak akkor, ha fájdalomcsillapítást alkalmazunk
- rutinszerűen, protokoll alapján
- rutinszerűen, de nincs protokoll az alkalmazásáról
- egyéb:

Ki végzi a fájdalomskála pontszámításait? (több válasz is megjelölhető)

- orvos
- nővér
- szülő
- egyéb

Rögzítik-e a dokumentációban a mért értéket?

- igen
- nem

Milyen gyakorisággal végzik a fájdalommérést? (több válasz adható)

- műszakonként legalább egyszer
- invazív beavatkozások előtt és után
- műtét után azonnal és óránként, amíg az analgézia optimálissá válik
- folyamatos fájdalomcsillapítás alkalmazásakor 4 óránként
- egyéb:

Melyik fájdalomskálát/skálákat használják?

Ha nem használnak fájdalomskálát, miért nem? (több válasz adható)

- nincs megfelelő skála
- túl időigényes
- túl bonyolult
- nem tartjuk fontosnak
- nincs értelme, enélkül is adekvátan csillapítjuk a fájdalmat
- a fájdalom megítélésénél más jelekre (fizikális, viselkedési) támaszkodunk
- más módszerrel (pl. NIRS, NIPE) mérjük a fájdalmat
- humánerőforrás hiány miatt
- egyéb:

Ha más módszerrel történik a fájdalommérés, mivel? (több válasz adható)

- NIRS
- amplitudóintegrált EEG
- NIPE monitor
- fiziológiai és/vagy viselkedési változások, jelek értékelése
- egyéb:

Ha nem fájdalomskálával, hanem fiziológiai és viselkedési változásokkal értékelik a fájdalmat, miket vizsgálnak? (több válasz adható)

- szívfrekvencia
- vérnyomás
- légzésszám
- oxigén szaturáció
- izomtónus
- sírás
- arckifejezés
- egyéb:

Használnak nem gyógyszeres fájdalomcsillapító módszereket?

- igen
- nem

Ha igen, melyiket használják fájdalomcsillapításra? (több válasz adható)

- oralis szacharóz oldat
- szoptatás
- lefejt anyatej
- cumi/nem tápláló célú szoptatás
- kenguruzás/bőr-bőr kontaktus
- masszázs
- multiszenzoros stimuláció
- zeneterápia
- aromaterápia
- ikrek ápolásánál co-bedding
- facilitated tucking/pozícionálás
- egyéb:

Ha használnak nem gyógyszeres fájdalomcsillapítást, azt jelzik az ápolási dokumentációban?

- igen
- nem

Ha igen, mely nem gyógyszeres fájdalomcsillapító módszert jelzik az ápolási dokumentációban?

- oralis szacharóz oldat
- szoptatás
- lefejt anyatej
- cumi/nem tápláló célú szoptatás
- kenguruzás/bőr-bőr kontaktus
- masszázs
- multiszenzoros stimuláció
- zeneterápia
- aromaterápia
- ikrek ápolásánál co-bedding
- facilitated tucking/pozícionálás
- egyéb:

Ha nem használnak nem gyógyszeres fájdalomcsillapítást, miért nem? (több válasz adható)

- mindig gyógyszeres fájdalomcsillapítást alkalmazunk, ha szükséges
- nem tartjuk hatékonynak
- nem tartjuk fontosnak
- nincs rá lehetőségünk
- elfelejtjük használni
- a rövid beavatkozásoknál, a beavatkozás rövidsége miatt nincs értelme használni
- egyéb:

Intubált, lélegeztetett beteg esetén használnak rutinszerűen fájdalomcsillapítást a lélegeztetéshez?

- igen
- nem

Ha igen, mit? (több válasz adható)

- morfin folyamatos infúzió
- morfin bólus
- fentanyl folyamatos infúzió
- fentanyl bólus
- nalbuphin folyamatos infúzió
- nalbuphin bólus
- ketamin
- egyéb:

Alkalmaznak rutinszerűen posztoperatív fájdalomcsillapítást?

- igen
- nem
- nem látunk el posztoperatív eseteket

Ha igen, mit? (több válasz adható)

- morfin folyamatos infúzióban
- morfin bólus
- fentanyl folyamatos infúzió
- fentanyl bólus
- nalbuphin folyamatos infúzió
- nalbuphin bólus
- ketamin
- paracetamol
- ibuprofen
- egyéb:

A folyamatos posztoperatív fájdalomcsillapításban részesülő beteg procedurális fájdalom (pl. vénaszúrás) esetén részesül-e extra fájdalomcsillapításban?

- igen
- nem
- nem látunk el posztoperatív eseteket

Használnak-e szedatívumot (pl. phenobarbital) önmagában fájdalomcsillapításra?

- igen
- nem

Ha igen, mit? (több válasz adható)

- benzodiazepint
- phenobarbitalt
- chloralhydratot
- egyéb:

Ön megfelelőnek tartja az osztályon a fájdalomcsillapítási gyakorlatot?

- igen
- nem
- nem rossz, de lehetne rajta javítani
- egyéb:

Ha van-bármilyen egyéb észrevétele, kommentje, megjegyzése a felméréssel és az osztályos fájdalomcsillapítási gyakorlattal kapcsolatban, kérem írja le! (önálló válasz)
